# Comparison of Long-Term Outcomes of Patients With Myocardia Infarction (MI) With Non-obstructive Coronary Arteries and MI With Obstructive Coronary Arteries: A Systematic Review and Meta-Analysis

**DOI:** 10.7759/cureus.43137

**Published:** 2023-08-08

**Authors:** Muhammad O Naeem, Saad Khalid Khan, Ramy R Gergess, Lokeshwar Raaju Addi Palle, Santoshi Krupanagaram, Muhammad Waqas Khan, Madiha D Haseeb, Shamsha Hirani

**Affiliations:** 1 Pathology, Rehman Medical Institute, Peshawar, PAK; 2 Medicine, Army Medical College, Rawalpindi, PAK; 3 Internal Medicine, Universidad Autónoma de Guadalajara, Guadalajara, MEX; 4 Surgery, Kamala Hospital, Chennai, IND; 5 General Surgery, Hackensack Meridian Health Palisades Medical Center, North Bergen, USA; 6 Medicine, MNR Medical College and Hospital, Sangareddy, IND; 7 Medicine, Services Institute of Medical Sciences, Lahore, PAK; 8 Neurology, Dow University of Health Sciences, Karachi, PAK; 9 Cardiology, Baqai Hospital, Karachi, PAK

**Keywords:** mioca, minoca, systematic review and meta-analysis, all-cause mortality, cardiovascular outcomes, myocardial infarction with obstructive coronary arteries, myocardial infarction with non-obstructive coronary arteries (minoca)

## Abstract

The aim of this study was to compare long-term outcomes in patients with myocardial infarction with non-obstructive coronary arteries (MINOCA) and patients with myocardial infarction with obstructive coronary arteries (MIOCA). This meta-analysis was conducted according to the recommendations of the Preferred Reporting Items for Systematic Reviews and Meta-Analyses (PRISMA). The literature search was conducted in online databases including PubMed and Web of Science from 2010 onwards. Primary outcomes assessed in this meta-analysis included major adverse cardiovascular events (MACE) and all-cause mortality. Secondary outcomes included cardiovascular mortality and myocardial infarction. A total of 16 studies were included in the meta-analysis. Pooled analysis showed that the risk of MACE was higher in MIOCA patients (risk ratio (RR): 1.47, 95%CI: 1.43-1.52, p-value: 0.001) compared to MINOCA patients. Additionally, the risk of all-cause mortality was also significantly higher in MIOCA patients compared to MINOCA (RR: 1.33, 95%CI: 1.14-1.56, p-value: 0.001). Our findings also indicate that patients with MIOCA are at a significantly higher risk of recurrent myocardial infarction and cardiovascular-related mortality compared to patients with MINOCA. Overall, the insights gained from this meta-analysis have significant clinical implications, guiding decision-making in the management of patients with MINOCA.

## Introduction and background

Around 5-10% of individuals diagnosed with myocardial infarction (MI) have no significant blockages in their coronary arteries [1-2]. In these cases, the condition is referred to as MI with non-obstructive coronary arteries (MINOCA). MINOCA can occur due to various causes such as the disruption of coronary plaques, spasms, blood clots, artery tearing, impaired microcirculation, or myocardial injury caused by an imbalance between oxygen supply and demand [3-4]. MINOCA represents a diverse and varied disease state. The significance of MINOCA has been recently highlighted in the European guidelines for managing ST-segment elevation MI [5]. Evaluating the risk in patients with MINOCA poses difficulties due to the varied causes underlying the condition, which differ from those observed in patients with MI with obstructive coronary artery disease (MIOCA). Limited and conflicting data exist regarding the factors that predict MINOCA and its long-term outcomes [6-7]. However, it is becoming more evident that MINOCA is not uncommon, especially among individuals who experience an early-onset MI.

Evidence from observational studies and systematic reviews suggests that MINOCA patients have a higher likelihood of experiencing negative outcomes [8-9]. However, there is a scarcity of data specifically examining cardiovascular morbidity in MINOCA [10], and to the best of our knowledge, cause-specific mortality has not been studied. Obtaining such information is crucial for gaining a deeper understanding of the potential disease mechanisms that differentiate MINOCA from MIOCA. It can also help in tailoring patient management strategies based on these distinctions [11].

In terms of prognosis, a systematic review identified a small number of studies revealing a 12-month all-cause death rate of 6.7% in MIOCA patients compared with 3.5% in MINOCA patients [12]. However, recent data on outcomes in MINOCA patients have been limited mainly to mortality. There is a scarcity of data related to the clinical profile and health status of these patients. The recognition of MINOCA as a separate and distinct condition highlights the importance of gaining a thorough understanding of its prognosis, including the risk of cardiovascular events and so on. It is crucial to acquire comprehensive knowledge about the likely outcomes and long-term outlook for MINOCA patients. Therefore, there is a need for an updated meta-analysis to understand the long-term outcomes in patients with MINOCA and MIOCA. Therefore, this meta-analysis has been conducted to compare long-term outcomes in both sets of patients.

## Review

Materials and methods

This meta-analysis was conducted according to the recommendations of the Preferred Reporting Items for Systematic Reviews and Meta-Analyses (PRISMA). The literature search was conducted through the PubMed and Web of Science databases from 2010 onwards. Additionally, we also searched Google Scholar to find any additional article relevant to study objective. The following key search terms were included in different combinations: "ST-elevation myocardial infarction" OR "non-ST segment elevation myocardial infarction" AND "obstructive coronary atherosclerosis" AND "non-obstructive coronary atherosclerosis" OR "mild coronary artery disease," "insignificant coronary artery disease" OR "significant coronary artery disease" AND "death" OR "all-cause death" OR "all-cause mortality" OR "mortality" OR "cardiac death" OR "death from cardiovascular disease" OR "myocardial infarction" OR "reinfarction" OR "MACE" OR "major adverse cardiovascular events" OR "stroke." We also used Medical Subject Headings (MeSH) terms to further sensitize the search. In addition, bibliographic data of included studies were also manually searched to identify undiscovered studies during the initial phase of searching. We restricted our search to only those studies that were published in the English language.

Study Selection and Quality Assessment

All records were initially screened by two authors independently using their titles and abstracts, followed by a detailed assessment of the full text based on pre-defined inclusion and exclusion criteria. We included articles that enrolled patients with MINOCA or MIOCA at baseline. MINOCA was characterized as the absence of any epicardial vessel with stenosis equal to or greater than 50%, as determined by quantitative coronary angiography. Non-obstructive lesions were further categorized into two groups: mild coronary stenosis (with 1-49% lumen stenosis in at least one vessel) and normal coronary vessels (with 0% lumen stenosis in all vessels). Studies that reported one of the required outcomes were included in the meta-analysis. We excluded studies that included patients with types of acute coronary syndrome (ACS) other than MI. Lastly, we excluded studies with a follow-up of fewer than 12 months.

Quality assessment of included studies was done by two authors using the Newcastle-Ottawa Scale (NOS) comparative research quality assessment system. Studies were deemed to be of high quality if they achieved a score of six stars or more out of a total of nine stars on the NOS scale. Any disagreements between the two authors in the process of study selection and quality assessment were resolved via discussion or involvement of a third author if required.

Data Extraction and Outcome Measures

Data were extracted from included studies using a standardized data extraction form. The data extracted from included studies included author names, year of publication, study design, sample size, follow-up duration, and patients' characteristics. If outcomes were reported at multiple time points, the last available outcomes were extracted. Primary outcomes assessed in this meta-analysis included major adverse cardiovascular events (MACE) and all-cause mortality. Secondary outcomes included cardiovascular mortality and MI.

Data Analysis

To compare the outcomes between study groups, risk ratio (RR) was computed with a 95% confidence interval (CI) using a fixed or random effect model based on the value of I-square. The cut-off for p-value was kept at 0.05. Heterogeneity was computed using I-square and Cochran-Q tests. A p-value < 0.1 was considered significant for heterogeneity. Publication bias was assessed using Egger's test. We performed meta-regression to understand the causes of heterogeneity among the study outcomes. Data analysis was performed using ReviewManager (RevMan) version 5.4.1 (The Cochrane Collaboration, London, United Kingdom) and Stata version 16.0 (2019; StataCorp LLC, College Station, Texas, United States).

Results

**Figure 1:**
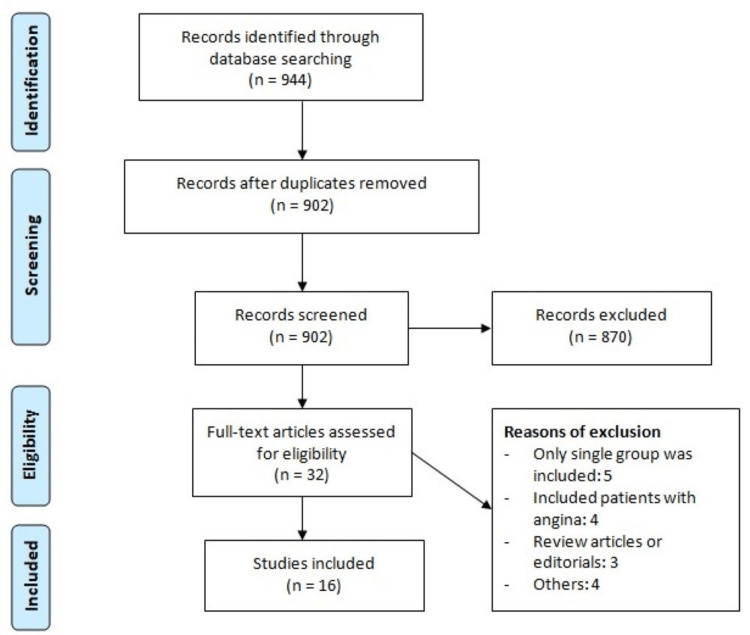
PRISMA flowchart of study selection PRISMA: Preferred Reporting Items for Systematic Reviews and Meta-Analyses

Figure 1 shows the process of study selection. Online database searching yielded 944 studies. After removing duplicates, 902 records were initially screened using their titles and abstracts. Full texts of 32 articles were obtained, and a detailed evaluation was done based on pre-defined inclusion and exclusion criteria. Finally, 16 studies were included in the present meta-analysis. Table 1 shows the characteristics of the included studies. The follow-up of included studies ranged from 12 to 74 months. Table 2 presents the quality assessment of included studies.

**Table 1:** Characteristics of included studies MIOCA: myocardial infarction with obstructive coronary arteries; MINOCA: myocardial infarction with non-obstructive coronary arteries

Author and Reference	Year	Region	Study Design	Study Group	Sample Size	Follow-up
Abdu et al. [13]	2019	China	Prospective cohort	MIOCA	1730	12 Months
				MINOCA	109	
Bainey et al. [14]	2018	Canada	Retrospective cohort	MIOCA	33836	12 Months
				MINOCA	2092	
Barr et al. [15]	2017	New Zealand	Retrospective cohort	MIOCA	1768	28.8 Months
				MINOCA	302	
Choo et al. [16]	2019	Korea	Prospective cohort	MIOCA	10871	24 Months
				MINOCA	396	
Dreyer et al. [17]	2020	United States	Retrospective cohort	MIOCA	269931	12 Months
				MINOCA	16849	
Eggers et al. [18]	2018	Sweden	Retrospective cohort	MIOCA	69267	45.6 Months
				MINOCA	7266	
Gasior et al. [19]	2020	Poland	Retrospective cohort	MIOCA	160866	36 Months
				MINOCA	6063	
Juan-Salvadores et al. [20]	2022	Spain	Retrospective cohort	MIOCA	243	74 Months
				MINOCA	32	
Kang et al. [21]	2011	Korea	Retrospective cohort	MIOCA	2930	12 Months
				MINOCA	126	
Lopez-Pais et al. [22]	2020	Spain	Prospective cohort	MIOCA	412	17.3 Months
				MINOCA	109	
Magnani et al. [23]	2021	Italy	Prospective cohort	MIOCA	1671	19.9 Months
				MINOCA	313	
Monteiro et al. [24]	2022	Portugal	Retrospective cohort	MIOCA	2015	60 Months
				MINOCA	428	
Planer et al. [25]	2014	United States	Retrospective cohort	MIOCA	2245	12 Months
				MINOCA	197	
Rhew et al. [26]	2012	Korea	Retrospective cohort	MIOCA	1120	13 Months
				MINOCA	100	
Safdar et al. [27]	2018	United States	Prospective cohort	MIOCA	2374	12 Months
				MINOCA	299	
Williams et al. [28]	2019	New Zealand	Retrospective cohort	MIOCA	7408	24 Months
				MINOCA	897	

**Table 2:** Quality assessment of included studies

Author and Reference	Selection	Exposure	Outcome	Total
Abdu et al. [13]	3	2	3	Good
Bainey et al. [14]	3	2	4	Good
Barr et al. [15]	3	2	3	Good
Choo et al. [16]	2	2	3	Good
Dreyer et al. [17]	3	2	3	Good
Eggers et al. [18]	3	1	3	Good
Gasior et al. [19]	2	2	3	Good
Juan-Salvadores et al. [20]	2	1	3	Fair
Kang et al. [21]	3	2	4	Good
Lopez-Pais et al. [22]	3	2	3	Good
Magnani et al. [23]	3	2	3	Good
Monteiro et al. [24]	2	2	3	Good
Planer et al. [25]	3	2	2	Good
Rhew et al. [26]	3	1	3	Good
Safdar et al. [27]	3	2	4	Good
Williams et al. [28]	2	1	3	Fair

Comparison of Baseline Characteristics of Patients in MINOCA and MIOCA Groups

As shown in Table 3, the number of males in the MIOCA group is lower compared to the MINOCA group. Patients with MINOCA are less likely to be diabetic, hypertensive, and have dyslipidemia. Additionally, the likelihood of being diagnosed with ST elevation MI was also lower among MINOCA patients compared to their counterparts.

**Table 3:** Baseline characteristics of MIOCA and MINOCA patients RR: risk ratio; STEMI: ST-elevation myocardial infarction ^ represented as mean difference  (95% CI) MIOCA: myocardial infarction with obstructive coronary arteries; MINOCA: myocardial infarction with non-obstructive coronary arteries

Variable	Number of Studies	RR (95% CI)	I-square
Male	16	0.54 (0.49-0.59)	98%
Hypertension	14	1.08 (1.02-1.14)	80%
Diabetes	14	1.38 (1.23-1.54)	92%
STEMI	12	2.12 (1.52-2.96)	99%
Dyslipidemia	9	1.21 (1.05-1.38)	93%
Age	13	7.25 (8.10, 6.40)^	85%

Comparison of Cardiovascular Outcomes

**Figure 2:**
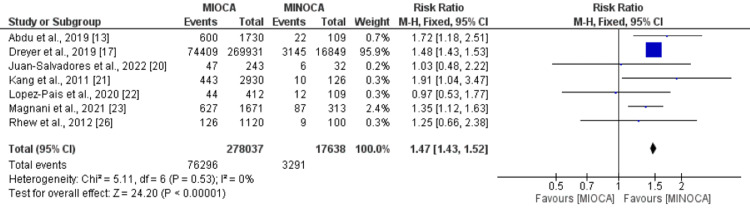
Major adverse cardiovascular events References: [13,17,20-23,26] MIOCA: myocardial infarction with obstructive coronary arteries; MINOCA: myocardial infarction with non-obstructive coronary arteries

MACE: Seven studies were included in the pooled analysis of MACE. As shown in Figure 2, the risk of MACE was significantly higher in patients with MIOCA compared to patients with MINOCA (RR: 1.47, 95%CI: 1.43-1.52, p-value: 0.001). No significant heterogeneity was reported among the study results (I-square: 0%). Most of the weight is carried by the study conducted by Dreyer et al., potentially due to a large sample size. Therefore, we performed a sensitivity analysis by removing this study, and the effect estimate was similar to the overall analysis (RR: 1.30, 95%CI: 1.19-1.61, p-value: 0.001).

**Figure 3:**
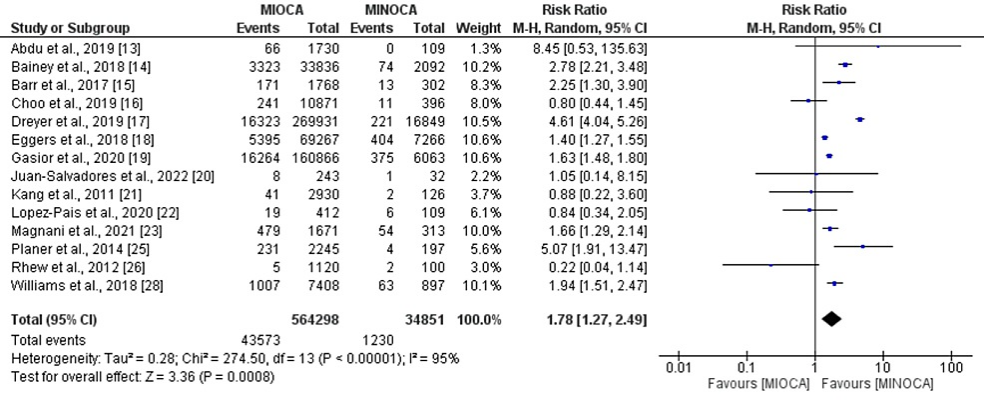
Recurrent myocardial infarction Sources: References [13-23,25-26,28] MIOCA: myocardial infarction with obstructive coronary arteries; MINOCA: myocardial infarction with non-obstructive coronary arteries

**Figure 4:**
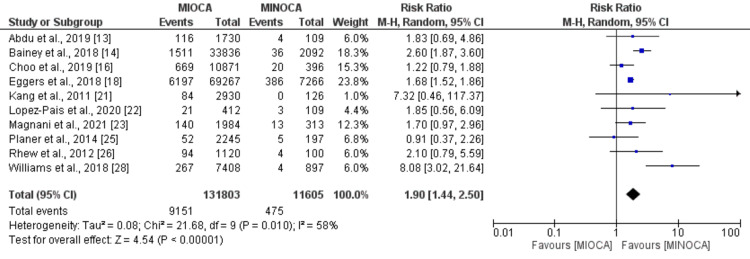
Cardiovascular-related mortality Sources: References [13-14,16,18,21-23,25-26,28] MIOCA: myocardial infarction with obstructive coronary arteries; MINOCA: myocardial infarction with non-obstructive coronary arteries

Recurrent MI and cardiovascular-related mortality: The pooled analysis of 14 studies comparing the risk of MI between patients with MIOCA and MINOCA showed that the risk of myocardial infarction was significantly higher in patients with MIOCA compared to MINOCA (RR: 1.78, 95% CI: 1.27 to 2.49, p-value: 0.001), as shown in Figure 3. Significant heterogeneity was reported among the study results (I-square: 95%). Regarding cardiovascular-related mortality, the pooled analysis of 10 studies showed that the risk of cardiovascular events was significantly higher in patients with MIOCA compared to patients with MINOCA (RR: 1.90, 95%CI: 1.44 to 2.50, p-value: 0.001), as shown in Figure 4. Significant heterogeneity was reported among the study results (I-square: 58%).

All-cause Mortality

**Figure 5:**
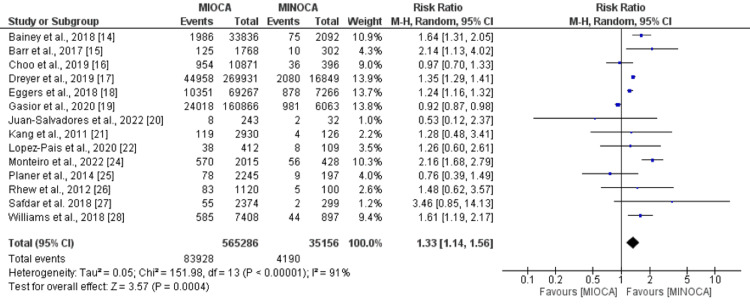
All-cause mortality References: [14-22,24-28] MIOCA: myocardial infarction with obstructive coronary arteries; MINOCA: myocardial infarction with non-obstructive coronary arteries

Pooled analysis of 14 studies comparing the risk of all-cause mortality between MIOCA and MINOCA patients reported that the risk of all-cause mortality was 1.33 times higher in MIOCA patients compared to patients with MINOCA (RR: 1.33, 95%CI: 1.14-1.56, p-value: 0.001) as shown in Figure 5. Significant heterogeneity was reported among the study result (I-square: 91%).

Meta-Regression

To explore possible sources of heterogeneity in the pooled analysis of primary outcomes, which included MACE and all-cause mortality, we performed a meta-regression by assessing the impact of follow-up period, age, male gender, hypertension, STEMI, diabetes, aspirin, beta-blockers, statin, and renin-angiotensin (RAS) inhibitors. The results are shown in Table 4. Regarding MACE, meta-regression identified diabetes, female gender, and dyslipidemia as potential sources of heterogeneity. However, for all-cause mortality, meta-regression identified STEMI, diabetes, and female gender as potential sources of heterogeneity among the study results. No indication of publication bias was found, with Egger’s tests being statistically non-significant in all of the outcomes assessed in the present study (p-value>0.05).

**Table 4:** Meta-regression STEMI: ST-elevation myocardial infarction; MACE: major adverse cardiovascular events; RAS: renin-angiotensin

Variable	MACE	All-cause death
Follow-up	0.755	0.898
Age	0.451	0.399
Female	0.021	0.041
Hypertension	0.033	0.858
Diabetes	0.802	0.006
STEMI	0.747	0.028
Dyslipidemia	0.016	0.554
RAS	0.658	0.458
Statins	0.644	0.723
Bblockers	0.484	0.864
Aspirin	0.155	0.395

Discussion

The present meta-analysis reanalyzed all the data published related to the clinical outcomes of MIOCA and MINOCA patients, attempting to provide quantitative estimates of their long-term outcomes. The pooled analysis showed that the risk of MACE was higher in MI patients with coronary artery disease (CAD) compared to MI patients without CAD. In a separate analysis of the individual components of MACE, we found that the risk of all-cause mortality, cardiac mortality, and recurrent MI were higher in MIOCA subjects compared to the MINOCA patients.

In the majority of the included studies, MINOCA patients showed better long-term outcomes compared to MIOCA patients. Additionally, a lower rate of mortality was also reported in a recent systematic review that included patients with MINOCA [12]. The most plausible reasons for these observations are likely linked to the younger age and lower prevalence of diabetes mellitus among MINOCA individuals, both of which independently predict a lower risk of MACE. Furthermore, due to the significantly reduced occurrence of ST-segment elevation-ACS during baseline presentation in MINOCA patients, it is possible that their average extent of MI is smaller compared to MIOCA subjects [29]. The present meta-analysis has also confirmed these findings, as the number of individuals with diabetes is lower in MINOCA patients compared to MIOCA. Additionally, the number of STEMI patients was also significantly lower in MINOCA subjects compared to their counterparts.

The superior baseline coronary heart disease (CHD) risk profile of MINOCA compared to MIOCA individuals has been extensively studied and documented in numerous research works. These studies have proposed various potential explanations, focusing on factors related to the progression of atherosclerotic plaque and highlighting the possible stronger influence of non-classical risk factors (such as inflammation, insulin resistance, psychosocial factors, and physical inactivity) in the etiology of ACS for MINOCA subjects [30-31]. This meta-analysis now provides precise quantitative estimates with narrow confidence intervals on the prevalence of the most common CHD risk factors in both MIOCA and MINOCA groups. These findings have practical implications as they can be utilized in clinical practice or as support for developing prognostic multivariate models.

Considering that the underlying mechanisms that lead to the clinical presentation of MI in MIOCA patients are not completely understood and that the relevant therapeutic approach for these patients is also not known [32-33], it has been proposed that their unfavorable prognosis could be attributed, at least partially, to the reduced prescription rate of medications such as beta-blockers, angiotensin-converting enzyme inhibitors, statins, and antiplatelet drugs [33-34]. To confirm these findings, future studies need to be carried out to assess the impact of these drugs on the prognosis in patients with MINOCA.

Study Limitations

Several limitations should be considered in the interpretation of our data. First, the heterogeneity across studies was substantial in both the baseline characteristics and the length of follow-up. Secondly, most studies were retrospective in nature and retrospective studies might have limited external validity. The study populations, settings, and conditions under investigation may not fully represent the broader population or real-world scenarios. Therefore, more prospective studies need to be conducted to validate the findings. Furthermore, the planned meta-regression analyses conducted to examine variations between studies lacked the necessary strength to detect connections between variables, as they only relied on aggregated data from each study. We do not have individual-level data to understand the impact of different variables on prognosis. Therefore, future studies need to be conducted to assess the effect of different variables such as comorbidities, medications, and laboratory characteristics on prognosis.

## Conclusions

This comprehensive meta-analysis provides valuable insights into the long-term outcomes of patients with MINOCA and MIOCA. Our findings indicate that patients with MIOCA are at a significantly higher risk of MACE, recurrent MI, cardiovascular-related mortality, and all-cause mortality compared to patients with MINOCA. Overall, the insights gained from this meta-analysis have significant clinical implications, guiding decision-making in the management of patients with MINOCA. It is our hope that this study will provide a foundation for future research aimed at improving the outcomes of these patients and tailoring their treatment strategies effectively.

## References

[1] Reynolds HR (2012). Myocardial infarction without obstructive coronary artery disease. Curr Opin Cardiol.

[2] Agewall S, Beltrame JF, Reynolds HR (2017). ESC working group position paper on myocardial infarction with non-obstructive coronary arteries. Eur Heart J.

[3] Scalone G, Niccoli G, Crea F (2019). Editor's choice- pathophysiology, diagnosis and management of MINOCA: an update. Eur Heart J Acute Cardiovasc Care.

[4] Khan A, Lahmar A, Riasat M (2022). Myocardial Infarction with non-obstructive coronary arteries: an updated overview of pathophysiology, diagnosis, and management. Cureus.

[5] Ibanez B, James S, Agewall S (2018). 2017 ESC guidelines for the management of acute myocardial infarction in patients presenting with ST-segment elevation: the task force for the management of acute myocardial infarction in patients presenting with ST-segment elevation of the European Society of Cardiology (ESC). Eur Heart J.

[6] Tamis-Holland JE, Jneid H, Reynolds HR (2019). Contemporary diagnosis and management of patients with myocardial infarction in the absence of obstructive coronary artery disease: a scientific statement from the American Heart Association. Circulation.

[7] Patel MR, Chen AY, Peterson ED (2006). Prevalence, predictors, and outcomes of patients with non-ST-segment elevation myocardial infarction and insignificant coronary artery disease: results from the can rapid risk stratification of unstable angina patients suppress adverse outcomes with early implementation of the ACC/AHA guidelines (CRUSADE) initiative. Am Heart J.

[8] Sluchinski SL, Pituskin E, Bainey KR, Norris CM (2020). A review of the evidence for treatment of myocardial infarction with nonobstructive coronary arteries. CJC Open.

[9] Lindahl B, Baron T, Albertucci M, Prati F (2021). Myocardial infarction with non-obstructive coronary artery disease. EuroIntervention.

[10] Ciliberti G, Coiro S, Tritto I (2018). Predictors of poor clinical outcomes in patients with acute myocardial infarction and non-obstructed coronary arteries (MINOCA). Int J Cardiol.

[11] Crea F, Montone RA, Niccoli G (2020). Myocardial infarction with non-obstructive coronary arteries: dealing with pears and apples. Eur Heart J.

[12] Pasupathy S, Air T, Dreyer RP, Tavella R, Beltrame JF (2015). Systematic review of patients presenting with suspected myocardial infarction and nonobstructive coronary arteries. Circulation.

[13] Abdu FA, Liu L, Mohammed AQ (2019). Myocardial infarction with non-obstructive coronary arteries (MINOCA) in Chinese patients: clinical features, treatment and 1 year follow-up. Int J Cardiol.

[14] Bainey KR, Welsh RC, Alemayehu W (2018). Population-level incidence and outcomes of myocardial infarction with non-obstructive coronary arteries (MINOCA): Insights from the Alberta contemporary acute coronary syndrome patients invasive treatment strategies (COAPT) study. Int J Cardiol.

[15] Barr PR, Harrison W, Smyth D, Flynn C, Lee M, Kerr AJ (2018). Myocardial infarction without obstructive coronary artery disease is not a benign condition (ANZACS-QI 10). Heart Lung Circ.

[16] Choo EH, Chang K, Lee KY (2019). Prognosis and predictors of mortality in patients suffering myocardial infarction with non‐obstructive coronary arteries. J Am Heart Assoc.

[17] Dreyer RP, Tavella R, Curtis JP (2020). Myocardial infarction with non-obstructive coronary arteries as compared with myocardial infarction and obstructive coronary disease: outcomes in a Medicare population. Eur Heart J.

[18] Eggers KM, Hjort M, Baron T, Jernberg T, Nordenskjöld AM, Tornvall P, Lindahl B (2019). Morbidity and cause-specific mortality in first-time myocardial infarction with nonobstructive coronary arteries. J Intern Med.

[19] Gasior P, Desperak A, Gierlotka M (2020). Clinical characteristics, treatments, and outcomes of patients with myocardial infarction with non-obstructive coronary arteries (MINOCA): results from a multicenter national registry. J Clin Med.

[20] Juan-Salvadores P, Jiménez Díaz VA, Rodríguez González de Araujo A (2022). Clinical features and long-term outcomes in very young patients with myocardial infarction with non-obstructive coronary arteries. J Interv Cardiol.

[21] Kang WY, Jeong MH, Ahn YK (2011). Are patients with angiographically near-normal coronary arteries who present as acute myocardial infarction actually safe?. Int J Cardiol.

[22] Lopez-Pais J, Izquierdo Coronel B, Galán Gil D (2022). Clinical characteristics and prognosis of myocardial infarction with non-obstructive coronary arteries: a prospective single-center study. Cardiol J.

[23] Magnani G, Bricoli S, Ardissino M (2022). Long-term outcomes of early-onset myocardial infarction with non-obstructive coronary artery disease (MINOCA). Int J Cardiol.

[24] Monteiro E, Barbosa J, Guimaraes J (2022). Comparing the long-term prognosis of myocardial infarction with non-obstructive coronary arteries to myocardial infarction with obstructive coronary artery disease. Eur Heart J.

[25] Planer D, Mehran R, Ohman EM, White HD, Newman JD, Xu K, Stone GW (2014). Prognosis of patients with non-ST-segment-elevation myocardial infarction and nonobstructive coronary artery disease: propensity-matched analysis from the Acute Catheterization and Urgent Intervention Triage Strategy trial. Circ Cardiovasc Interv.

[26] Rhew SH, Ahn Y, Kim MC (2012). Is myocardial infarction in patients without significant stenosis on a coronary angiogram as benign as believed?. Chonnam Med J.

[27] Safdar B, Spatz ES, Dreyer RP (2018). Presentation, clinical profile, and prognosis of young patients with myocardial infarction with nonobstructive coronary arteries (MINOCA): results from the VIRGO study. J Am Heart Assoc.

[28] Williams MJ, Barr PR, Lee M, Poppe KK, Kerr AJ (2019). Outcome after myocardial infarction without obstructive coronary artery disease. Heart.

[29] Sun J, Zhang W, Zeng Q, Dong S, Sun X (2012). Three-year follow-up in patients with acute coronary syndrome and normal coronary angiography. Coron Artery Dis.

[30] Larsen AI, Galbraith PD, Ghali WA, Norris CM, Graham MM, Knudtson ML (2005). Characteristics and outcomes of patients with acute myocardial infarction and angiographically normal coronary arteries. Am J Cardiol.

[31] Roe MT, Harrington RA, Prosper DM (2000). Clinical and therapeutic profile of patients presenting with acute coronary syndromes who do not have significant coronary artery disease.The platelet glycoprotein IIb/IIIa in unstable angina: receptor suppression using integrilin therapy (PURSUIT) trial investigators. Circulation.

[32] Niccoli G, Scalone G, Crea F (2015). Acute myocardial infarction with no obstructive coronary atherosclerosis: mechanisms and management. Eur Heart J.

[33] Saleh M, Ambrose JA (2018). Understanding myocardial infarction. F1000Res.

[34] Rossini R, Capodanno D, Lettieri C (2013). Long-term outcomes of patients with acute coronary syndrome and nonobstructive coronary artery disease. Am J Cardiol.

